# Supplementary material for: Nonlinear ion-acoustic waves with Landau damping in non-Maxwellian space plasmas
Source: Sci Rep. 2024 Jun 6;14:13005. doi: 10.1038/s41598-024-63773-7 (PMC11156942; doi:10.1038/s41598-024-63773-7)
Supplement: Supplementary file 2 — Supplementary Information 2. [file 41598_2024_63773_MOESM2_ESM.pdf]

Supplemental Material: Derivation of Eq. (23)

Using the variable stretching Ansatz Eq. (13), we have: $\frac{\partial}{\partial x} = \varepsilon^{\frac{1}{2}} \frac{\partial}{\partial \xi}$ and $\frac{\partial}{\partial t} = -V\varepsilon^{\frac{1}{2}} \frac{\partial}{\partial \xi} + \varepsilon^{\frac{3}{2}} \frac{\partial}{\partial \tau}$.

Leading-order perturbation $\varepsilon^{\frac{3}{2}}$:

Equating the coefficients of $\varepsilon^{\frac{3}{2}}$ from Eq. (6)-(8) and Eq. (12)

$$-V\frac{\partial n^{(1)}}{\partial \xi} + \frac{\partial u^{(1)}}{\partial \xi} = 0\,, \tag{39}$$

$$-V\frac{\partial u^{(1)}}{\partial \xi} + \frac{\partial \phi^{(1)}}{\partial \xi} = 0\,, \tag{40}$$

$$n_e^{(1)} - n^{(1)} = 0\,, \tag{41}$$

and from the Vlasov equation,

$$v\frac{\partial f^{(1)}}{\partial \xi} + \frac{\partial \phi^{(1)}}{\partial \xi}\frac{\partial f^{(0)}}{\partial v} = 0\,, \tag{42}$$

where v denotes the microscopic velocity of the electrons. After integrating Eqs. (39) and (40), we obtain:

$$n^{(1)} = \frac{1}{V^2}\phi^{(1)} \qquad \text{and} \qquad u^{(1)} = \frac{1}{V}\phi^{(1)}\,. \tag{43}$$

To obtain a unique solution of Eq. (42), we shall include ad hoc in Eq. (42) a higher-order term originating from the third-order expressions in ε, i.e.[13]

$$\gamma_1\varepsilon^2\frac{\partial f_\varepsilon^{(1)}}{\partial \tau} + v\frac{\partial f_\varepsilon^{(1)}}{\partial \xi} + \frac{\partial \phi^{(1)}}{\partial \xi}\frac{\partial f_\varepsilon^{(0)}}{\partial v} = 0\,. \tag{44}$$

The solutions of the initial value problems (44) can now be found uniquely, once $f_\varepsilon^{(1)}$ is known, by letting $\varepsilon \to 0$ as: $f^{(1)} = \lim_{\varepsilon\to 0} f_\varepsilon^{(1)}$. Now, taking the Fourier transform of the above equation,

$$f(\omega,k) = \int_{-\infty}^{\infty}\int_{-\infty}^{\infty} f(\xi,\tau)\exp i(k\xi - \omega\tau)\,d\xi\,d\tau\,, \tag{45}$$

we obtain

$$f^{(1)} = \lim_{\varepsilon\to 0}\left(\frac{-k\frac{\partial f^{(0)}}{\partial v}}{kv - \omega\gamma_1\varepsilon^2}\right)\phi^{(1)}\,. \tag{46}$$

Recalling the identity

$$\lim_{\varepsilon\to 0}\left(\frac{1}{k\omega - \omega\gamma_1\varepsilon^2}\right) = P\left(\frac{1}{kv}\right) + i\pi\,\delta(kv)\,, \tag{47}$$

where P denotes the Cauchy principal value and δ is Dirac's delta function, we can rewrite Eq. (46) as:

$$f^{(1)} = -k\frac{\partial f^{(0)}}{\partial v}\left[P\left(\frac{1}{kv}\right) + i\pi\delta(kv)\right]\phi^{(1)} = -2\frac{\partial f^{(0)}}{\partial v^2}\phi^{(1)}\,. \tag{48}$$

From Eqs. (4) and (19), we obtain:

$$n_e^{(1)} = \int_{-\infty}^{+\infty} -2\frac{\partial}{\partial v^2}\left[\frac{1}{\sqrt{\pi}\theta}\frac{\Gamma(\kappa+1)}{\kappa^{\frac{3}{2}}\Gamma(\kappa-\frac{1}{2})}\left(1+\frac{v^2}{\kappa\theta^2}\right)^{-\kappa}\right]\phi^{(1)}dv = \left(\frac{\kappa-\frac{1}{2}}{\kappa-\frac{3}{2}}\right)\phi^{(1)} = a_1\phi^{(1)}\,. \tag{49}$$

Combining Eqs. (43) and (49) into (41), we get

$$a_1\phi^{(1)} - \frac{1}{V^2}\phi^{(1)} = 0\,.$$

The phase speed is thus prescribed as

$$V = \frac{1}{\sqrt{a_1}}\,, \qquad \text{where} \qquad a_1 = \left(\frac{\kappa-\frac{1}{2}}{\kappa-\frac{3}{2}}\right).$$

Higher-order perturbation $\varepsilon^{\frac{5}{2}}$

In order $\varepsilon^{\frac{5}{2}}$, from Eqs. (6)-(8) and (12), we have:

$$\frac{\partial n^{(1)}}{\partial \tau} - \gamma_2 V \frac{\partial n^{(2)}}{\partial \xi} + \gamma_2 \frac{\partial u^{(2)}}{\partial \xi} + \gamma_2 \frac{\partial n^{(1)} u^{(1)}}{\partial \xi} = 0, \tag{50}$$

$$\frac{\partial u^{(1)}}{\partial \tau} - \gamma_2 V \frac{\partial u^{(2)}}{\partial \xi} + \gamma_2 u^{(1)} \frac{\partial u^{(1)}}{\partial \xi} + \gamma_2 \frac{\partial \phi^{(2)}}{\partial \xi} = 0, \tag{51}$$

$$\frac{\lambda_D^2}{L^2} \frac{\partial^2 \phi^{(1)}}{\partial \xi^2} = \gamma_2 (n_e^{(2)} - n^{(2)}), \tag{52}$$

and (53)

$$n_e^{(2)} = \int_{-\infty}^{+\infty} f^{(2)} dv. \tag{54}$$

The Vlasov equation in this order reads:

$$-\gamma_1 \gamma_2 V \frac{\partial f^{(1)}}{\partial \xi} + v \gamma_2^2 \frac{\partial f^{(2)}}{\partial \xi} + \gamma_2^2 \frac{\partial \phi^{(1)}}{\partial \xi} \frac{\partial f^{(1)}}{\partial v} + \gamma_2^2 \frac{\partial \phi^{(2)}}{\partial \xi} \frac{\partial f^{(0)}}{\partial v} = 0,$$

i.e.

$$v \frac{\partial f^{(2)}}{\partial \xi} + \frac{\partial \phi^{(2)}}{\partial \xi} \frac{\partial f^{(0)}}{\partial v} = \frac{\gamma_1}{\gamma_2} V \frac{\partial f^{(1)}}{\partial \xi} - \frac{\partial \phi^{(1)}}{\partial \xi} \frac{\partial f^{(1)}}{\partial v}. \tag{55}$$

Now, introducing Eq. (49) into Eq. (55),

$$v \frac{\partial f^{(2)}}{\partial \xi} + \frac{\partial \phi^{(2)}}{\partial \xi} \frac{\partial f^{(0)}}{\partial v} = -2 \frac{\gamma_1}{\gamma_2} V \frac{\partial \phi^{(1)}}{\partial \xi} \frac{\partial f^{(0)}}{\partial v^2} + 2\phi^{(1)} \frac{\partial \phi^{(1)}}{\partial \xi} \frac{\partial}{\partial v} \frac{\partial f^{(0)}}{\partial v^2},$$

i.e.

$$v \frac{\partial f^{(2)}}{\partial \xi} + \frac{\partial \phi^{(2)}}{\partial \xi} \frac{\partial f^{(0)}}{\partial v} = -2 \frac{\gamma_1}{\gamma_2} V \frac{\partial \phi^{(1)}}{\partial \xi} \frac{\partial f^{(0)}}{\partial v^2} + 2\phi^{(1)} \frac{\partial \phi^{(1)}}{\partial \xi} \left(\frac{dv^2}{dv} \right) \frac{\partial^2 f^{(0)}}{\partial (v^2)^2},$$

and finally

$$v \frac{\partial f^{(2)}}{\partial \xi} + \frac{\partial \phi^{(2)}}{\partial \xi} \frac{\partial f^{(0)}}{\partial v} = -2 \frac{\gamma_1}{\gamma_2} V \frac{\partial \phi^{(1)}}{\partial \xi} \frac{\partial f^{(0)}}{\partial v^2} + 4v\phi^{(1)} \frac{\partial \phi^{(1)}}{\partial \xi} \frac{\partial^2 f^{(0)}}{\partial (v^2)^2}. \tag{56}$$

Now, let us set

$$C_1 = \frac{\gamma_1}{\gamma_2} V \frac{\partial \phi^{(1)}}{\partial \xi}, \qquad \text{and} \qquad D_1 = \phi^{(1)} \frac{\partial \phi^{(1)}}{\partial \xi}.$$

We can write Eq. (56) as

$$v \frac{\partial f^{(2)}}{\partial \xi} + \frac{\partial \phi^{(2)}}{\partial \xi} \frac{\partial f^{(0)}}{\partial v} = -2C_1 \frac{\partial f^{(0)}}{\partial v^2} + 4vD_1 \frac{\partial^2 f^{(0)}}{\partial (v^2)^2}.$$

Now, proceeding as above to get a unique solution, we introduce a higher order term to the above equation, viz.

$$\gamma_1 \varepsilon^2 \frac{\partial f_\varepsilon^{(2)}}{\partial \tau} + v \frac{\partial f_\varepsilon^{(2)}}{\partial \xi} + \frac{\partial \phi^{(2)}}{\partial \xi} \frac{\partial f_\varepsilon^{(0)}}{\partial v} = -2C_1 \frac{\partial f_\varepsilon^{(0)}}{\partial v^2} + 4vD_1 \frac{\partial^2 f_\varepsilon^{(0)}}{\partial (v^2)^2}. \tag{57}$$

Taking the Fourier transform of the latter equation, we obtain

$$f_\varepsilon^{(2)} = \left[\frac{-k \frac{\partial f^{(0)}}{\partial v}}{kv - \omega \gamma_1 \varepsilon^2} \right] \phi^{(2)} - 2i \lim_{\varepsilon \to 0} \left[\frac{C_1 \frac{\partial f^{(0)}}{\partial v^2} + 2vD_1 \frac{\partial^2 f^{(0)}}{\partial (v^2)^2}}{kv - \omega \gamma_1 \varepsilon^2} \right].$$

To obtain a unique solutios, we need to find $f^{(2)}$ by letting $\varepsilon \to 0$ in the above equation as $f^{(2)} = \lim_{\varepsilon\to 0} f_\varepsilon^{(2)}$.

Taking the Fourier transform of Eq. (57), we find

$$f^{(2)} = \lim_{\varepsilon\to 0}\left[\frac{-k\frac{\partial f^{(0)}}{\partial v}}{kv-\omega\gamma_1\varepsilon^2}\right]\phi^{(2)} - 2i\lim_{\varepsilon\to 0}\left[\frac{C_1\frac{\partial f^{(0)}}{\partial v^2}+2vD_1\frac{\partial^2 f^{(0)}}{\partial (v^2)^2}}{kv-\omega\gamma_1\varepsilon^2}\right],$$

i.e.

$$f^{(2)} = -k\frac{\partial f^{(0)}}{\partial v}\left[P\left(\frac{1}{kv}\right)+i\pi\delta(kv)\right]\phi^{(2)} - 2i\left[C_1\frac{\partial f^{(0)}}{\partial v^2}+2vD_1\frac{\partial^2 f^{(0)}}{\partial (v^2)^2}\right]\left[P\left(\frac{1}{kv}\right)+i\pi\delta(kv)\right],$$

and

$$f^{(2)} + a_1\phi^{(2)} = 2i\left[C_1\frac{\partial f^{(0)}}{\partial v^2}+2vD_1\frac{\partial^2 f^{(0)}}{\partial (v^2)^2}\right]\left[P\left(\frac{1}{kv}\right)+i\pi\delta(kv)\right].$$

Multiplying the above equation by ik and integrating over v, we have

$$ik[n_e^{(2)} - a_1\phi^{(2)}] = 2C_1\int_{-\infty}^{+\infty}\frac{\partial f^{(0)}}{\partial v^2}(i\pi k\delta(kv))dv + 4D_1\int_{\infty}^{+\infty}\frac{\partial^2 f^{(0)}}{\partial (v^2)^2}(kvP(kv))\,dv. \tag{58}$$

Substituting with the kappa distribution function[23]

$$f^{(0)} = \frac{1}{\sqrt{\pi}\theta}\frac{\Gamma(\kappa+1)}{\kappa^{\frac{3}{2}}\Gamma(\kappa-\frac{1}{2})}\left(1+\frac{v^2}{\kappa\theta^2}\right)^{-\kappa} \qquad \text{with} \qquad \theta = \left(\frac{2\kappa-3}{\kappa}\right)^{\frac{1}{2}},$$

one finds

$$\int_{-\infty}^{+\infty}\frac{\partial^2 f^{(0)}}{\partial (v^2)^2}dv = \frac{1}{4}\frac{\left(\kappa^2-\frac{1}{4}\right)}{\left(\kappa-\frac{3}{2}\right)^2} \qquad \text{and} \qquad \int_{-\infty}^{+\infty}k\frac{\partial f^{(0)}}{\partial v^2}\delta(kv)dv = \frac{1}{2\sqrt{2\pi}}\frac{\Gamma(\kappa+1)}{(\kappa-\frac{3}{2})^{\frac{3}{2}}\Gamma(\kappa-\frac{1}{2})}.$$

We take the inverse-Fourier transform of Eq. (58) and substituting the values of C_1 and D_1, we find

$$\frac{\partial n_e^2}{\partial\xi} - a_1\frac{\partial\phi^{(2)}}{\partial\xi} = a_3\phi^{(1)}\frac{\partial\phi^{(1)}}{\partial\xi} + a_2F^{-1}\left(i\,\mathrm{sgn}(k)\right)C_1,$$

where

$$F^{-1}\left(i\,\mathrm{sgn}(k)\right) = -\left(\frac{1}{\pi}\right)P\left(\frac{1}{\xi}\right).$$

Here, we have defined the quantities:

$$a_1 = \left(\frac{\kappa-\frac{1}{2}}{\kappa-\frac{3}{2}}\right), \qquad a_2 = \frac{\gamma_1 V}{\sqrt{2\pi}}\frac{\Gamma(\kappa+1)}{(\kappa-\frac{3}{2})^{\frac{3}{2}}\Gamma(\kappa-\frac{1}{2})}, \qquad \text{and} \qquad a_3 = \frac{\left(\kappa^2-\frac{1}{4}\right)}{\left(\kappa-\frac{3}{2}\right)^2}, \tag{59}$$

while $\mathrm{sgn}(k) = k/|k|$ is the sign of k.

Using the convolution theorem

$$P\left(\frac{1}{\xi}\right)\frac{\partial\phi^{(1)}}{\partial\xi} = P\int_{-\infty}^{+\infty}\left(\frac{\partial\phi^{(1)}}{\partial\xi'}\right)\frac{d\xi'}{\xi-\xi'}, \tag{60}$$

we obtain

$$\frac{\partial n_e^2}{\partial\xi} - a_1\frac{\partial\phi^{(2)}}{\partial\xi} + \frac{a_2}{\gamma_2}P\int_{-\infty}^{+\infty}\left(\frac{\partial\phi^{(1)}}{\partial\xi'}\right)\frac{d\xi'}{\xi-\xi'} - a_3\phi^{(1)}\frac{\partial\phi^{(1)}}{\partial\xi} = 0. \tag{61}$$

Multiplying Eq. (50) by V and adding to Eq. (51),

$$\frac{\partial u^{(1)}}{\partial \tau}+V\frac{\partial n^{(1)}}{\partial \tau}+\gamma_2 u^{(1)}\frac{\partial u^{(1)}}{\partial \xi}+\gamma_2\frac{\partial \phi^{(2)}}{\partial \xi}-\gamma_2 V^2\frac{\partial n^{(2)}}{\partial \xi}+\gamma_2 V\frac{\partial n^{(1)}u^{(1)}}{\partial \xi}=0\,. \tag{62}$$

Differentiating both sides of Eq. (52) (i.e. operating by $\frac{\partial}{\partial \xi}$),

$$\gamma_3\frac{\partial^3 \phi^{(1)}}{\partial \xi^3}=\gamma_2\left(\frac{\partial n_e^{(2)}}{\partial \xi}-\frac{\partial n^{(2)}}{\partial \xi}\right),$$

i.e.

$$\gamma_2\frac{\partial n^{(2)}}{\partial \xi}=\gamma_2\frac{n_e^{(2)}}{\partial \xi}-\gamma_3\frac{\partial^3 \phi^{(1)}}{\partial \xi^3}\,. \tag{63}$$

Now, combining Eq. (63) into Eq. (62),

$$\frac{\partial u^{(1)}}{\partial \tau}+V\frac{\partial n^{(1)}}{\partial \tau}+\gamma_2 u^{(1)}\frac{\partial u^{(1)}}{\partial \xi}+\gamma_2\frac{\partial \phi^{(2)}}{\partial \xi}-V^2\gamma_2\frac{n_e^{(2)}}{\partial \xi}+\gamma_3 V^2\frac{\partial^3 \phi^{(1)}}{\partial \xi^3}+\gamma_2 V\frac{\partial n^{(1)}u^{(1)}}{\partial \xi}=0\,.$$

Substituting $\frac{\partial n_e^2}{\partial \xi}$ from Eq. (61),

$$\frac{\partial u^{(1)}}{\partial \tau}+V\frac{\partial n^{(1)}}{\partial \tau}+\gamma_2 u^{(1)}\frac{\partial u^{(1)}}{\partial \xi}+\gamma_2\frac{\partial \phi^{(2)}}{\partial \xi}-V^2\gamma_2\left[a_1\frac{\partial \phi^{(2)}}{\partial \xi}-\frac{a_2}{\gamma_2}P\int_{-\infty}^{+\infty}\left(\frac{\partial \phi^{(1)}}{\partial \xi'}\right)\frac{\partial \xi'}{\xi-\xi'}+a_3\phi^{(1)}\frac{\partial \phi^{(1)}}{\partial \xi}\right]$$
$$+\gamma_3 V^2\frac{\partial^3 \phi^{(1)}}{\partial \xi^3}+\gamma_2 V\frac{\partial n^{(1)}u^{(1)}}{\partial \xi}=0.$$

One may now eliminate $\frac{\partial u^{(2)}}{\partial \xi}$ and $\frac{\partial n^{(2)}}{\partial \xi}$ by using Eqs. (50)- (52) and then substituting the values of $\phi^{(1)}$ and $u^{(1)}$ from Eqs. (43):

$$\phi^{(1)}=V^2 n^{(1)} \qquad \text{and} \qquad u^{(1)}=Vn^{(1)}\,.$$

One thus obtains

$$2V\frac{\partial n^{(1)}}{\partial \tau}+\gamma_2 V^2 n^{(1)}\frac{\partial n^{(1)}}{\partial \xi}+a_2 V^4 P\int_{-\infty}^{+\infty}\left(\frac{\partial n^{(1)}}{\partial \xi'}\right)\frac{d\xi'}{\xi-\xi'}-a_3 V^6\gamma_2 n^{(1)}\frac{\partial n^{(1)}}{\partial \xi}+\gamma_3 V^4\frac{\partial^3 n^{(1)}}{\partial \xi^3}+2\gamma_2 V^2 n^{(1)}\frac{\partial n^{(1)}}{\partial \xi}=0\,,$$

and

$$2V\frac{\partial n^{(1)}}{\partial \tau}+3\gamma_2 V^2 n^{(1)}\frac{\partial n^{(1)}}{\partial \xi}+a_2 V^4 P\int_{-\infty}^{+\infty}\left(\frac{\partial n^{(1)}}{\partial \xi'}\right)\frac{d\xi'}{\xi-\xi'}-a_3 V^6\gamma_2 n^{(1)}\frac{\partial n^{(1)}}{\partial \xi}+\gamma_3 V^4\frac{\partial^3 n^{(1)}}{\partial \xi^3}=0\,,$$

or

$$\frac{\partial n^{(1)}}{\partial \tau}+\frac{\gamma_2}{2V}\left[3V^2-a_3V^6\right]n^{(1)}\frac{\partial n^{(1)}}{\partial \xi}+\frac{a_2V^4}{2V}P\int_{-\infty}^{+\infty}\left(\frac{\partial n^{(1)}}{\partial \xi'}\right)\frac{d\xi'}{\xi-\xi'}+\frac{\gamma_3 V^3}{2}\frac{\partial^3 n^{(1)}}{\partial \xi^3}=0\,.$$

The latter expression is a modified KdV equation (with an extra term in account of Landau damping) in the form:

$$\frac{\partial n}{\partial \tau}+An\frac{\partial n}{\partial \xi}+B\frac{\partial^3 n}{\partial \xi^3}+CP\int_{-\infty}^{+\infty}\left(\frac{\partial n}{\partial \xi'}\right)\frac{d\xi'}{\xi-\xi'}=0\,, \tag{64}$$

i.e. precisely Eq. (23).
